# Associations between 24 h Movement Behavior and Mental Health in Office Workers

**DOI:** 10.3390/ijerph17176214

**Published:** 2020-08-27

**Authors:** Lisa-Marie Larisch, Lena V. Kallings, Maria Hagströmer, Manisha Desai, Philip von Rosen, Victoria Blom

**Affiliations:** 1 Department for Physical Activity and Health, The Swedish School of Sport and Health Sciences, 114 33 Stockholm, Sweden; lena.kallings@gih.se (L.V.K.); victoria.blom@gih.se (V.B.); 2 Department of Public Health and Caring Sciences, Family Medicine and Preventive Medicine, Uppsala University, 752 36 Uppsala, Sweden; 3 Department of Neurobiology, Care sciences and Society, Division of Physiotherapy, Karolinska Institutet, 141 83 Stockholm, Sweden; maria.hagstromer@ki.se (M.H.); philip.von.rosen@ki.se (P.v.R.); 4 Academic Primary Health Care Centre, Region Stockholm, 113 65 Stockholm, Sweden; 5 Department of Health Promoting Science, Sophiahemmet University, 11486 Stockholm, Sweden; 6 Quantitative Science Unit, Stanford University, Palo Alto, CA 94304, USA; manishad@stanford.edu; 7 Department of Neuroscience, Karolinska Institutet, 171 65 Stockholm, Sweden

**Keywords:** 24 h movement behavior, compositional data analysis, common mental health disorders, office workers

## Abstract

The associations between 24 h movement behavior, i.e., the way people distribute their time in different movement-related behaviors, on mental health are not well understood. This study applied a compositional data analysis approach to explore cross-sectional associations between device-measured moderate to vigorous physical activity (MVPA), light intensity physical activity (LIPA), sedentary behavior (SED), self-reported time in bed and mental health outcomes, i.e., depression or anxiety symptoms, burnout, mental wellbeing and stress, in office workers. ActiGraph accelerometers were worn for 24 h for at least 4 days to assess MVPA, LIPA, and SED. Sleep diaries were used in addition to identify time in bed. Analytic sample sizes for the different outcomes ranged from N = 345–370 participants. In this population of office workers with high levels of MVPA, the entire movement behavior composition was not associated to any of the mental health outcomes, but MVPA relative to all other behaviors was positively associated with mental wellbeing. This confirms the importance of MVPA for health relative to other movement-related behaviors.

## 1. Introduction

Mental ill health poses a heavy individual, social, and economic burden. In 2016, approximately 84 million people—that is one in six people—across the countries in the European Union were estimated to have a mental health problem, based on a variety of data sources and estimates [1]. Reliability and comparability of data across countries however is limited due to varying levels of stigma and awareness surrounding mental health problems as well as availability of mental health services [1].

In Sweden, for example, psychiatric diagnoses have been the most common reason for sickness absence since 2014 [2]. Stress-related and mood disorders account for 90% of all sickness absence cases due to psychiatric disorders [3].

Previous studies have shown that time spent in different movement-related behaviors, such as physical activity (PA) at different intensities, sedentary behavior (SED), and sleep affect mental health. In sum, the existing literature indicates that PA has positive effects on mental health outcomes in the focus of this study, i.e., depression or anxiety symptoms, burnout, mental wellbeing, and stress [4,5,6,7,8,9,10,11,12,13]. In contrast, extensive SED may affect these mental health outcomes negatively [11,12], while both too long and too short sleep durations are associated with worse mental health [14,15].

However, studies have predominantly used self-reported measures of PA and SED [4,6,8,10,13,16], which are considered to be less valid and reliable compared to accelerometer measurements [17]. In addition, many previous studies focused on participation in structured exercise [4,8,10,13,16], not on habitual PA at different intensities accumulated throughout the whole day. Accelerometers overcome the challenges of recall and social desirability bias inherent to self-report measures and provide detailed information on how people spend their time in different movement-related behaviors throughout the 24 h continuum. Moreover, most previous studies considered different movement-related behaviors as independent risk factors, while they in fact are mutually exclusive parts of the 24 h continuum and affect health synergistically [18,19,20]. People’s daily time is constrained to 24 h, thus engaging in one behavior can only occur at the expense of other behaviors [21]. Therefore, they should not be analyzed and interpreted in isolation, but in relation to each other [20]. Understanding how people distribute their time as well as the mechanisms for the underlying choices [21], may improve lifestyle interventions aimed at optimizing these behaviors. The compositional data analysis (CoDA) approach enables studying the combined effects of different movement-related behaviors [22]. This composition of different movement-related behaviors will be referred to as 24 h movement behavior.

Few studies have analyzed the effects of 24 h movement behavior on mental health outcomes using CoDA [23,24,25,26,27]. One study did not find any associations between 24 h movement behavior and depression, anxiety, stress, and mental health-related quality of life [27]. Other studies found a beneficial association between relative time spent in moderate to vigorous physical activity (MVPA) and self-rated mental health in older adults, but not in younger or middle-aged adults [25]. Time spent in MVPA relative to sleep and study time was also beneficially associated with emotional exhaustion in students [24]. Replacing work time with PA or sleep when transitioning from work to retirement was associated with substantial reduction in depression, anxiety, and stress and small improvements in self-esteem and mental wellbeing [23]. Increases in SED relative to other behaviors were associated with increases in depressive symptoms among adults, and simulating a replacement of SED with MVPA or sleep, but not light-intensity physical activity (LIPA), led to significant, but small reductions in depressive symptoms [26]. These studies indicate that higher intensity behaviors have a positive effect on various mental health outcomes when their interplay with other behaviors is taken into account. However, one study used self-reported PA and did not differentiate between PA intensities [23], sleep has not been measured by devices in any of the studies, and one study did not consider all behaviors occurring throughout the day [24]. While these studies provide a starting point towards a better understanding of the combined effects of movement-related behaviors on mental health, more research is needed to understand how movement-related behaviors are related to mental health outcomes in different populations, taking the entire 24 h continuum into account and using objective measures of these behaviors.

Since psychiatric diagnoses are very common causes of sickness absence among the working population [2], the target group of this study was office workers, who represent a large proportion of the population. Office workers spend 71–82% of their working hours sitting and are thus at increased risk for accumulating large amounts of sedentary time during working hours [28,29,30]. A more comprehensive understanding of the associations between 24 h movement behavior and mental health outcomes can provide valuable information for occupational health interventions as well as for more comprehensive public health recommendations [31].

Consequently, the aims of this study were:

1. To investigate whether the entire 24 h movement behavior composition, consisting of MVPA, LIPA, SED, and time in bed, is associated with mental health outcomes, i.e., depression or anxiety symptoms, burnout, mental wellbeing, and stress in a population of healthy office workers.
2. To investigate associations between time spent in any of these movement-related behaviors, relative to the others, and the mental health outcomes.
3. To investigate theoretical effects of reallocating time between the behaviors.

## 2. Materials and Methods 

### 2.1. Participants

This study used data from two different projects from the “Physical activity and healthy brain functions” research project, including participants from the same two Swedish companies. Data were collected on mental health outcomes, sociodemographics, movement-related behaviors, and fitness in the same manner in both projects. All employees received oral and written information about the study and provided written informed consent. The projects were conducted in accordance with the Declaration of Helsinki. Eligible participants were at least 18 years old. Participants did not receive any compensation for their participation, but data collection took place at their workplace during working hours and they received individual written feedback on their PA, SED, and fitness after completion of data collection.

Project one was a cross-sectional study and data were collected in 2016–2017. Exclusion criteria were health complaints affecting the person’s ability to stand or walk and pregnancy. Ethical approval was granted by the Stockholm regional ethics committee (2016/1840-32).

**Figure 1:**
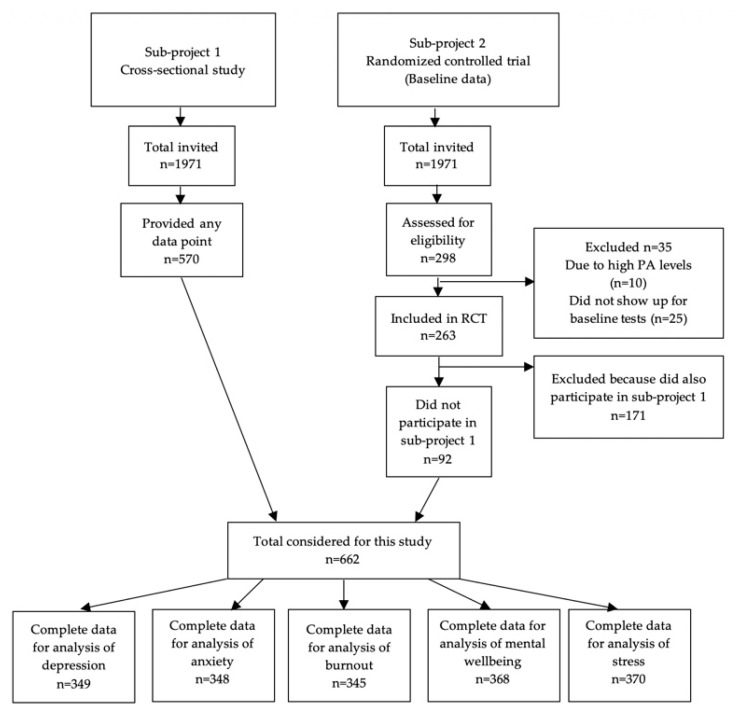
Flow-chart of the number of participants included from the two projects.

Project two was a randomized controlled trial (RCT) that is described elsewhere [32]. Baseline data of persons who did not participate in project one were included in this study. Data were collected in 2018. Persons with high PA levels, defined as more than 30 min/day spent in MVPA in bouts of at least 10 min, assessed via accelerometers, were not eligible for this project (N = 10) since the RCT targeted less-active persons. Ethical approval for the RCT was granted by the Stockholm regional ethical review board (2017/2409-31/1). In total, 662 participants (project one N = 570, project two N = 92) were considered for this study (see Figure 1).

### 2.2. Mental Health Outcomes

In this study, we investigated five different mental health outcomes: symptoms of depression, symptoms of anxiety, burnout, mental wellbeing, and stress. The following validated instruments were part of an online questionnaire that participants received via email around the same time as the accelerometer measurements were performed. In addition to evaluating mental health outcomes, the questionnaire included questions about sociodemographics, work stressors, life-style factors, and general health.

The Hospital Anxiety and Depression Scale (HAD) [33] is a 14-item questionnaire with two subscales used to assess anxiety and depression symptoms. Respondents are asked to recall how they have felt during the previous week. A global index is calculated for each condition with higher values indicating poorer mental health. Results are recommended to be categorized into normal (0–7), borderline abnormal (8–10), and abnormal (11–21) [33]. The Shirom–Melamed Burnout Measure consists of 14 items asking about the respondent’s emotional exhaustion, physical fatigue, and cognitive weariness [34]. A global index of burnout is calculated with higher values indicating poorer mental health (low burnout ≤ 2.75, high burnout ≥ 4.47). The WHO-Five well-being scale is a measure of mental wellbeing and reflects aspects other than just the absence of depressive symptoms (low mood though not necessarily depression ≤ 50, likely depression ≤ 28) [35]. The single-item measure of stress symptoms asks how often a person experienced stress during the past week on a scale from one (less than a few times per months or never) to five (every day) [36]. A mean score was calculated for stress.

### 2.3. Movement-Related Behaviors

We conceptualized the 24 h movement behavior composition as consisting of MVPA, LIPA, SED, and time in bed. MVPA, LIPA, and SED were assessed using ActiGraph^TM^ GT3X accelerometers (Actigraph LLC, Pensacola, FL, USA). Participants were instructed to wear the device for seven consecutive days on the right anterior superior iliac spine during wake time and around the left wrist using an elastic band when going to bed and to remove it only for water activities. Data were collected at a sampling frequency of 30 Hz.

Time in bed was derived from diaries, which participants were asked to fill out during the measurement period. Participants were asked to document the time when they started to try to fall asleep and out-of-bed times. Sleep researchers argue that the time it takes to fall asleep and awakenings after sleep onset are normal, often healthy parts of the sleep–wake cycle [37]. Therefore, we did not include periods identified as sleep per se as part of the composition, but rather conceptualized time in bed as sleep-related behavior [37].

To take sleep quality into account, which can affect health independently of sleep duration [38], we adjusted for objectively derived sleep efficiency (proportion of total time in bed actually spent asleep), derived through the ActiLife software (version 6.13.3) (ActiGraph LLC, Pensacola, FL, USA) [39]. A minimum of five valid nights was set for deriving sleep efficiency [40]. Nights were considered as valid if diary information for time in bed was available and if the accelerometer output during night hours showed a typical sleep pattern.

The ActiLife software was used to derive daily minutes (min) spent in different intensity categories during self-reported wake time, averaged over the entire measurement period. If no self-reported wake time was available in the diary, a standard wake time from 6:00–23:00 was used. The following customized cut-off points for adults were used for categorizing vector magnitude activity counts into intensity levels: 1–200 counts per min (cpm) SED [41], 200–2689 cpm LIPA, and ≥2690 cpm MVPA [42]. Non-wear time during wake hours was defined as ≥60 consecutive min of zero counts, with allowance for maximum 2 min of non-zero counts. Accelerometer data were included in the analysis if participants had 600 min of valid wake wear time on at least four days [43].

### 2.4. Covariates

Sociodemograhic characteristics considered in this study, i.e., age, sex, and education, were assessed via an online questionnaire. Estimate maximal oxygen uptake (VO_2_ max) as a measure of cardiorespiratory fitness was assessed via the revised Ekblom-Bak test, a sub-maximal cycle ergometer test [44], and was expressed as relative values (mL per minute per kg body mass).

### 2.5. Statistical Analysis

For each mental health outcome, a sub-sample was created containing only those participants that had complete data for movement-related behavior, covariates, and the respective mental health outcome, thus leading to five different analytic sub-samples (see Figure 1). There were no significant differences between those with complete data and those with missing data for movement-related behavior, covariates, and the respective mental health outcome. Ordinary summary statistics (i.e., mean, standard deviation, and proportions) were used to describe the participants included in each analytic sub-sample on key demographic characteristics.

Compositional means of time spent in different movement-related behaviors (MVPA, LIPA, SED, time in bed) were calculated by creating the geometric mean and rescaling those to sum to 1440 min. Each participants’ daily time use was expressed as a set of three isometric log ratio (*ilr*) coordinates, which map the compositions in real space and preserve all relative information about the four compositional parts (Appendix A).

First, we examined whether there was an association between the entire composition and each mental health outcome (global scores for depression symptoms, anxiety symptoms, burnout, mental wellbeing, and mean stress score) using the ANOVA type II test of deviance of the regressions (Aim 1). Subsequently, we analyzed whether each movement-related behavior relative to the other behaviors was significantly associated with the outcome (Aim 2). For this analysis, a set of three *ilr* coordinates was created for each movement-related behavior by rearranging the compositional parts. The set of three *ilr* coordinates for each behavior was then used as explanatory variables, with the first *ilr* coordinate containing all the relative information about this behavior, relative to the geometric mean of the remaining ones. Thus, one compositional linear regression model was conducted for each outcome with four different sets of *ilr* coordinates (three *ilr* coordinates for each movement-related behavior). Results are reported for the crude model, adjusted for age, sex, education (adjusted model), and additionally for fitness and sleep efficiency (fully adjusted model), with the results from the model adjusted for age, sex, and education considered as the main results. The distribution of residuals was inspected post-hoc to ensure that the normality assumption for linear regression was fulfilled. All tests were two-sided and conducted at the 0.05 level of significance. The five mental health outcomes were chosen a priori, and no multiplicity corrections were performed.

Whenever a significant relationship between a movement-related behavior and an outcome was observed, we explored how reallocating relative time spent in this behavior to and from another would affect the outcome, using compositional isotemporal substitution [45] (Aim 3). Time was exchanged between two behaviors at a time (one-for-one reallocation) while keeping the others constant at their geometric mean. The samples’ mean compositions were used as a reference or starting point. Each altered composition was then entered as a predictor in the regression model to predict the mental health outcome, adjusting for age, sex, and education. Confidence intervals (CI) were calculated for the predicted health outcome values to see whether they differed from the average outcome values. All analyses were conducted in R [46], CoDA analyses were performed using the *Compositions* package [47].

Since the outcome variable stress was a 5-point Likert scale variable, we treated the variable as an ordinal approximation of a continuous variable and performed linear regression [48]. As a sensitivity analysis, we modeled stress as a categorical variable in multinomial regression models.

We performed an additional explorative analysis to analyze the associations between relative time spent in different activities and mental wellbeing in more detail. For this analysis, we distinguished between moderate physical activity (MPA) and at least vigorous physical activity (at least VPA), thus constructing a composition consisting of at least VPA (VPA, ≥6167 cpm), MPA (MPA, 2690–6166 cpm), LIPA, SED, and time in bed. Following the same principles as described for the statistical analysis, we analyzed whether each movement-related behavior relative to the other behaviors was significantly associated with the outcome. A set of four *ilr* coordinates was created for each behavior by rearranging the compositional parts. The set of four *ilr* coordinates was then used as an explanatory variable, with the first *ilr* coordinate containing all the relative information about the respective movement-related behavior, relative to the geometric mean of the remaining ones. Thus, one compositional linear regression model was conducted for each outcome with five different sets of *ilr* coordinates (four *ilr* coordinates for each movement behavior).

## 3. Results

Out of 662 participants considered for this study, 444 participants participated in accelerometer measurements. Ninety-eight percent of these participants had valid accelerometer data (at least four days of valid wake time data and at least five nights of valid sleep data). There was valid accelerometer data from at least six days for 99.5% of participants. Average wake time behaviors (MVPA, LIPA, SED) and average total time in bed summed to 22.71 h per day. A total of 349 participants had complete data for analyzing associations of 24 h movement behavior with depression symptoms, 348 for anxiety symptoms, 345 for burnout, 370 for mental wellbeing, and 368 for stress (see Table 1).

Descriptive statistics for each sub-sample are presented in Table 1. Participants were predominantly female (68%), had a university or higher academic degree (68%), were aged 41 years on average (SD 9), and their fitness levels were similar to average reference values for adults aged 40–49 years (47.2 mL per kg body weight for men, 38.4 mL per kg body weight for women) [49]. Compositional means for the different movement-related behaviors were almost identical across analytic sub-samples: 62 min per day on average spent in MVPA (4%), 326 min (6 h) in LIPA (23%), 577 min (9.6 h) in SED (40%), and 475 min (7.9 h) in bed (33%).

The entire movement composition was not significantly associated with any of the outcomes (*p* > 0.05), see Table 2 (Aim 1).

Table 3 presents the linear regression model results of the relationships between the relative movement behaviors and mental health outcomes (Aim 2). Only time spent in MVPA, relative to all other behaviors, was significantly positively associated with mental wellbeing. However, this association was not significant after additionally adjusting for fitness and sleep efficiency (Table 3). Adjusted R^2^ values for the models and CI for the estimates can be found in Appendix B. Fitting and interpreting stress as a categorical variable in multinomial regression models was challenging due to the very large CI for some of the estimates.

**Figure 2:**
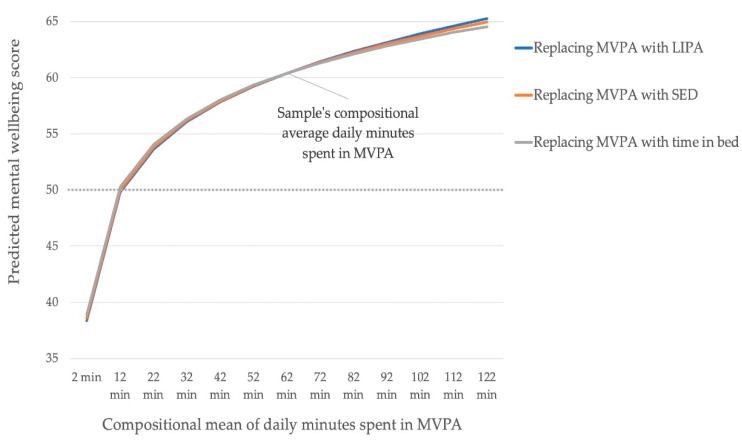
Predicted mental wellbeing scores for theoretically altered 24 h movement compositions. Time between MVPA and remaining behaviors were reallocated in steps of 10 min (one-for-one reallocations). Dashed line indicates cutoff between good mood and low mood (score of 50). MVPA: moderate to vigorous physical activity, LIPA: light intensity physical activity, SED: sedentary behavior.

Theoretically replacing the sample’s average 62 min per day in MVPA with LIPA, SED, or sleep in steps of 10 min revealed a non-linear reduction in mental wellbeing scores (Figure 2) (Aim 3). A table displaying the reallocation results can be found in Appendix C. An increase in relative time spent in MVPA was associated with an improved mental wellbeing score, and it did not matter whether that time was reallocated from LIPA, SED, or sleep. The associations were asymmetrical: The negative effects of reallocating time away from MVPA to other behaviors were greater than the benefits of increasing time in MVPA at the cost of other behaviors. When the average relative time in MVPA per day dropped below 12 min, the mental wellbeing score dropped below 50, the validated cut-off between good and low mood.

The linear regression models showed that the 24 h movement behavior composition explained a negligible variance for depression symptoms (adjusted R^2^ = −0.01), anxiety symptoms (adjusted R^2^ = 0.01), burnout (adjusted R^2^ = 0.005), mental wellbeing (adjusted R^2^ = 0.004), and stress (adjusted R^2^ = 0.003).

The additional explorative analysis where the composition of movement behaviors consisted of at least VPA, MPA, LIPA, SED, and time in bed revealed that relative time spent in at least VPA was significantly and positively associated with wellbeing, but not MPA. Results for this additional analysis can be found in Appendix D.

## 4. Discussion

This study investigated associations between the 24 h movement composition (consisting of MVPA, LIPA, SED, and time in bed) and five mental health outcomes, i.e., depression or anxiety symptoms, burnout, mental wellbeing, and stress. We found that the entire 24 h movement composition was not associated with any of the outcomes (Aim 1), but time spent in MVPA relative to all other behaviors was positively and significantly associated with mental wellbeing (Aim 2) when adjusting for age, sex, and education. An additional exploratory analysis in which MVPA was split up into MPA and at least VPA revealed that VPA relative to other behaviors was significantly associated with mental wellbeing.

Whereas the entire 24 h movement composition was not associated with mental wellbeing, relative time in MVPA was positively associated with mental wellbeing. This is explained by the fact that one joint *p*-value for the entire composition, expressed as a set of three *ilr* coordinates, was calculated when analyzing associations between the entire composition and mental wellbeing. However, when analyzing the association of MVPA relative to the remaining behaviors (LIPA, SED, time in bed), associations between each of the three *ilr* coordinates and the outcomes were calculated separately, with the first *ilr* coordinate containing the information regarding one behavior at a time in relation to all other behaviors. Thus, significant associations of relative time spent in one behavior relative to the remaining behaviors may or may not be found, regardless of whether or not the entire composition is significantly associated with an outcome. Thus, for the sample included in this study, it was only time spent in MVPA that was associated with mental wellbeing, irrespective of how time was distributed among the other behaviors.

Our findings are to some extent in line with previous studies. In a study with a comparable sample of adults [27], the entire 24 h movement composition (MVPA, LIPA, SED, and sleep) was not associated with depression, anxiety, stress, and mental health-related quality of life. Associations of relative time spent in different behaviors to the outcomes were not assessed. In another study, MVPA relative to sleep, SED, and LIPA was positively associated with self-rated mental health, but only in older adults (65–79 years), not in younger and middle-aged adults (18–64 years). The study did not investigate associations between the entire composition and outcomes. A study analyzing changes in time use across the retirement period found that changes in time use were associated with changes in depression, anxiety, stress, and self-esteem, but not mental wellbeing or life-satisfaction [23]. In this study. However, the time use composition consisted of self-reported time spent in nine different domains of everyday activities, with sleep and physical activity being two of them. Janurek et al. [24] found relative time spent in MVPA to be negatively associated with emotional exhaustion in undergraduate students, but the composition consisted of MVPA, study time, and sleep, thus results are difficult to compare. SED relative to sleep, LIPA, and MVPA was negatively associated with depressive symptoms in a large sample of adults in another study [26], associations of the other behaviors or the entire movement behavior composition with depression symptoms were not analyzed.

Our results suggest that more intense movement behaviors, MVPA and VPA, relative to the remaining behaviors, may be relevant for mental wellbeing but not for the other mental health outcomes. This may be explained by several factors. First, our sample consisted mainly of middle-aged, highly educated women that spend a high amount of daily time in MVPA (4.2%, 62 min) and were able to participate in work life. Results from population studies with samples representative of the general Swedish population showed that people spend on average 31 min per day in MVPA (women 29 min) [50]. However, these arithmetic means might differ slightly from the compositional means presented in this study. Participants in the study by Curtis et al. [28] had a comparable average composition of movement behaviors and they did not find any significant associations with depression, anxiety, or stress. Different results might be found for samples with less time spent in MVPA relative to other behaviors. One study with a sample that spent 1.88% (27 min) of the day on average in MVPA found SED to be associated with depression [26], compared to 4.2% (62 min) in our sample and 7% (107 min) in the study by Curtis et al., which did not find any associations with depression either [27]. Thus, it might be that as long as the proportion of MVPA is high relative to the other behaviors, it does not matter for mental wellbeing how time is distributed among the other behaviors.

Secondly, different results might be found for samples with poorer mental health than seen in our sample. A previous meta-analysis found that the effects of physical activity on depression may be stronger for clinical populations compared to non-clinical ones [51]. As del Pozo Cruz et al. [26] pointed out, continuous exposure to physical activity can reduce depression symptoms [51] and prevent the onset of depression in the general adult population [7]. It is likely that there are relationships between 24 h movement behavior and mental health outcomes that cannot be revealed by cross-sectional analyses in a healthy population. We cannot claim any causal inference or rule out reversed causality from the results of this study due to its cross-sectional design.

Reallocating time to or from MVPA was associated with relatively small differences in mental wellbeing scores. The mental wellbeing score dropped below 50, the cut-off between good mood and low mood, when MVPA was theoretically reduced to 12 min. This indicates that a certain amount of daily MVPA contributes to experiencing good mood, no matter how time is spent in other movement behaviors. Interestingly, the effects were the same for the different pairs of reallocations, meaning that it did not matter whether time was exchanged between MVPA and LIPA, MVPA and SED, or MVPA and time in bed. In another study with healthy adults with a smaller proportion of the day spent in MVPA, reallocating 60 min of SED with MVPA was associated with greater reductions in depression symptoms compared to replacing SED with sleep. However, the absolute reduction in depression symptoms was very small [26]. In line with a previous study [27], there was an asymmetry in effects: reducing time in MVPA had a greater negative impact on mental wellbeing than the positive impact of increasing MVPA by the same amount of time [27]. This finding in our study could also be explained by the fact that the proportion of MVPA per day was high and results might be different in populations with less MVPA.

In line with previous studies that used a CoDA approach to investigate associations between 24 h movement behavior and mental health outcomes, we found mostly small effect sizes [24,26,27,52] and that only a very small proportion of the variability in all mental health outcomes was explained by the 24 h movement composition when adjusting the regression models for age, sex, and education (adjusted R^2^ ranging from −0.01 to 0.01).

### Strengths and Limitations

Strengths of this study are that we included a range of common mental health disorders and used validated instruments. Furthermore, the analysis of time use covered the entire 24 h spectrum of device-measured movement-related behaviors, thus increasing the likelihood of capturing a valid representation of the participants’ movement behavior. Particular emphasis was put on the correct classification of time in bed rather than an often poorly defined period of sleep in previous studies. The CoDA approach allowed for a holistic analysis of the combined and synergistic effects of different movement behaviors, taking the compositional nature of time use data into account.

Several limitations have to be considered. While device-measured movement behavior provides a more reliable classification of time into intensity categories, it does not provide information on the type and context of activities performed, which can affect mental health positively or negatively. A widely accepted definition of SED is “any waking behavior characterized by an energy expenditure ≤1.5 metabolic equivalents (METs )while in a sitting or reclining posture” [53]. In this study however, we identified SED only based on the MET criteria and not posture. Thus, time spent in SED might have been overestimated and LIPA underestimated by classifying movement with ≤1.5 METs whilst standing as SED. Moreover, the duration of a bout in which a movement behavior is performed might determine the variation in different mental health outcomes [54]. More research is needed that can take these aspects into account. The study population consisted of mostly highly educated females with high levels of MVPA and generally good mental health, thus, results may not be representative of other populations.

## 5. Conclusions

Among, on average, middle-aged, mostly highly educated and female office workers with a large proportion of the day spent in MVPA, the entire 24 h movement behavior composition was not significantly associated with depression or anxiety symptoms, burnout, or stress. However, MVPA relative to LIPA, SED, and time in bed, was beneficially associated with mental wellbeing. Theoretically reducing MVPA to below 12 min per day, by replacing it with other behaviors, lead to a decline in mental wellbeing, from good mood to low mood. This confirms the importance of higher intensity movement behaviors for health. Future studies should examine the relationship of 24 h movement behaviors with mental health outcomes in populations with different distributions of movement behaviors across the day and different levels of mental health, using CoDA and longitudinal study designs.

This and further studies will be important contributions to the understanding of the relationship between 24 h movement behavior and mental health and to the development of 24 h movement guidelines for adults. Understanding how compositions of movement-related behaviors and single behaviors in relation to each other are associated with mental health outcomes may contribute to the design of successful behavior-change interventions aimed at helping people to find the right balance between movement behaviors throughout the day for maintaining or achieving good mental health.

## Figures and Tables

**Table 1:** Descriptive characteristics for each analytic sub-sample, containing only complete cases.

		Depression N = 349	Anxiety N = 348	Burnout N = 345	Mental Wellbeing N = 370	Stress N = 368
**Mental health outcomes**	Global score (mean ± SD)	3.2 ± 2.8	6.3 ± 3.7	2.0 ± 0.8	60.8 ± 17.8	2.7 ± 1.2
	N (%)	Normal: 320 (91.7%) Borderline abnormal: 21 (6%) Abnormal: 8 (2.3%)	Normal: 226 (64.9%) Borderline abnormal: 73 (21%) Abnormal: 49 (14.1%)	Healthy: 279 (80.9%) Low burnout: 64 (18.6%) High burnout: 2 (0.6%)	Good mood: 277 (74.9%) Low mood: 69 (18.6%) Likely depression: 24 (6.5%)	Less than a few times per month or never: 61 (16.6%) A few times per month: 116 (31.5%) Once per week: 86 (23.4%) Several times per week: 77 (20.9%) Every day: 28 (7.6%)
**Covariates**	Age (years) (mean ± SD)	41 ± 9	41 ± 9	41 ± 9	41 ± 9	41 ± 9
	Sex (male), N (%)	111 (32)	111 (32)	109 (32)	117 (32)	116 (32)
	Education, N (%) Primary school Gymnasium University or higher academic education	8 (2.3) 105 (30.1) 236 (67.6)	7 (2.0) 104 (29.9) 237 (68.1)	5 (1.4) 104 (30.1) 236 (68.4)	8 (2.2) 112 (30.3) 250 (67.6)	8 (2.2) 112 (30.4) 248 (67.4)
	Fitness (ml kg^−1^ min^−1^) (mean ± SD)	39.7 ± 8	39.8 ± 8	39.6 ± 8	39.5 ± 8	39.5 ± 8
	Sleep efficiency (mean ± SD)	90.2 ± 4	90.3 ± 4	90.3 ± 4	90.2 ± 4	90.2 ± 4
**Compositional mean for movement-related behaviors in minutes (% of 24 h)**	MVPA	62 (4%)	62 (4%)	61 (4%)	62 (4%)	62 (4%)
	LIPA	326 (22%)	326 (23%)	327 (23%)	327 (23%)	327 (23%)
	SED	577 (40%)	576 (40%)	576 (40%)	575 (40%)	575 (40%)
	Time in bed	475 (33%)	476 (33%)	475 (33%)	476 (33%)	476 (33%)

Note: for each mental health outcome, a sub-sample was created containing only those participants that had complete data for all variables of interest, thus leading to five different analytic sub-samples. MVPA: moderate to vigorous physical activity, LIPA: light intensity physical activity, SED: sedentary behavior.

**Table 2:** The *p*-values from the ANOVA type II test of deviance of the regression, examining the association of the entire 24 h movement composition with each mental health outcome.

Mental Health Outcomes	Crude Model	Adjusted Model	Fully Adjusted Model
Depression	0.867	0.873	0.627
Anxiety	0.574	0.717	0.635
Burnout	0.282	0.266	0.459
Mental wellbeing	0.126	0.105	0.261
Stress	0.653	0.869	0.837

Adjusted model includes the covariates age, sex, and education. Fully adjusted model additionally includes the covariates sleep efficiency and fitness.

**Table 3:** Results from linear regression models assessing associations between time spent in each movement-related behavior (time in bed, SED, LIPA, MVPA), relative to the remaining behaviors, and mental health outcomes.

Mental Health Outcomes	Crude Model				Adjusted Model				Fully Adjusted Model			
	MVPA	LIPA	SED	Time in Bed	MVPA	LIPA	SED	Time in Bed	MVPA	LIPA	SED	Time in Bed
**Depression**	−0.02 (0.964)	0.40 (0.621)	−0.66 (0.520)	0.29 (0.834)	0.003 (0.995)	0.43 (0.593)	-0.61 (0.567)	0.17 (0.904)	0.18 (0.721)	0.71 (0.377)	−0.76 (0.478)	−0.13 (0.926)
**Anxiety**	−0.03 (0.966)	1.0 (0.350)	−1.28 (0.343)	0.31 (0.866)	−0.16 (0.799)	1.14 (0.286)	−0.53 (0.703)	−0.45 (0.807)	−0.08 (0.899)	1.30 (0.227)	−0.57 (0.684)	−0.645 (0.727)
**Burnout**	−0.23 (0.094)	−0.07 (0.769)	−0.24 (0.389)	0.54 (0.163)	−0.25 (0.065)	−0.04 (0.851)	−0.13 (0.661)	0.42 (0.280)	−0.20 (0.148)	0.02 (0.944)	−0.21 (0.472)	0.39 (0.312)
**Mental wellbeing**	7.01 * **(0.019)**	−3.69 (0.460)	−1.61 (0.793)	−1.72 (0.838)	7.1 * **(0.018)**	−3.72 (0.458)	−3.66 (0.561)	0.28 (0.974)	5.48 (0.0668	−6.14 (0.217)	−0.49 (0.938)	1.15 (0.891)
**Stress**	0.10 (0.614)	−0.06 (0.851)	−0.46 (0.262)	0.42 (0.454)	0.10 (0.621)	−0.07 (0.834)	−0.27 (0.517)	0.24 (0.670)	0.12 (0.553)	−0.03 (0.930)	−0.29 (0.50)	0.20 (0.734)

* Statistically significant associations are shown in bold and denoted by asterisks (*p* < 0.05). Adjusted model includes the covariates age, sex, education. Fully adjusted model additionally includes the covariates sleep efficiency and fitness. Table shows the beta coefficients and *p*-values only for the first isometric log ratio coordinate that describes time spent in a specific behavior, relative to time in the remaining behaviors. MVPA: moderate to vigorous physical activity, LIPA: light intensity physical activity, SED: sedentary behavior.

## Appendix A

Creation of the isometric log ratio coordinates

(A1) $$ilr_{1}\;=\sqrt{\frac{3}{4}}\ln\left(\frac{MVPA}{LIPA*SED*Time\;in\;bed}\right)$$

(A2) $$ilr_{2}\;=\sqrt{\frac{2}{3}}\ln\left(\frac{LIPA}{SED*Time\;in\;bed}\right)$$

(A3) $$ilr_{3}\;=\sqrt{\frac{1}{2}}\ln\left(\frac{SED}{Time\;in\;bed}\right)$$

(A4) $$ilr_{1}\;=\sqrt{\frac{3}{4}}\ln\left(\frac{LIPA}{MVPA*SED*Time\;in\;bed}\right)$$

(A5) $$ilr_{2}\;=\sqrt{\frac{2}{3}}\ln\left(\frac{MVPA}{SED*Time\;in\;bed}\right)$$

(A6) $$ilr_{3}\;=\sqrt{\frac{1}{2}}\ln\left(\frac{SED}{Time\;in\;bed}\right)$$

(A7) $$ilr_{1}\;=\sqrt{\frac{3}{4}}\ln\left(\frac{SED}{LIPA*MVPA*Time\;in\;bed}\right)$$

(A8) $$ilr_{2}\;=\sqrt{\frac{2}{3}}\ln\left(\frac{LIPA}{MVPA*Time\;in\;bed}\right)$$

(A9) $$ilr_{3}\;=\sqrt{\frac{1}{2}}\ln\left(\frac{MVPA}{Time\;in\;bed}\right)$$

(A10) $$ilr_{1}\;=\sqrt{\frac{3}{4}}\ln\left(\frac{Time\;in\;bed}{LIPA*MVPA*SED}\right)$$

(A11) $$ilr_{2}\;=\sqrt{\frac{2}{3}}\ln\left(\frac{LIPA}{MVPA*SED}\right)$$

(A12) $$ilr_{3}\;=\sqrt{\frac{1}{2}}\ln\left(\frac{MVPA}{SED}\right)$$

## Appendix B

Beta coefficients, 95% CI and adjusted R^2^ values for the linear regression models investigating associations between time spent in one movement-related behavior (relative to the remaining ones) and mental health outcomes.

Respective tables display only the p-value for the first *ilr* coordinate that contains all relative information about the respective behavior, relative to the geometric mean of the remaining ones. Note that adjusted R^2^ values are the same across the models for each behavior. Adjusted model includes covariates age, sex, education. Fully adjusted model additionally includes covariates sleep efficiency and fitness. MVPA: moderate to vigorous physical activity, LIPA: light intensity physical activity, SED: sedentary behavior.

**Table A1:** Depression.

Movement-Related Behavior	Crude Model	Adjusted Model	Fully Adjusted Model
	β (95% CI)	β (95% CI)	β (95% CI)
**MVPA**	−0.02 (−0.98; 0.93)	0.003 (−0.96; 0.97)	0.18 (−0.79; 1.14)
**LIPA**	0.40 (−1.18; 1.97)	0.43 (−1.16; 2.02)	0.71 (−0.87; 2.30)
**SED**	−0.66 (−2.69; 1.36)	−0.61 (−2.69; 1.47)	−0.76 (−2.86; 1.340
**Time in bed**	0.29 (−2.42; 3.0)	0.17 (−2.59; 2.93)	−0.13 (−2.88; 2.62)

Adjusted R^2^ values for the crude model was −0.007, for the adjusted model −0.014 and for the fully adjusted model 0.007.

**Table A2:** Anxiety.

Movement-Related Behavior	Crude Model	Adjusted Model	Fully Adjusted Model
	β (95% CI)	β (95% CI)	β (95% CI)
**MVPA**	−0.03 (−1.23; 1.24)	−0.16 (−1.42; 1.10)	−0.08 (−1.36; 1.20)
**LIPA**	1.0 (−1.10; 3.10)	1.14 (−0.96; 3.24)	1.3 (−0.81; 3.41)
**SED**	−1.28 (−3.95; 1.38)	−0.53 (−3.23; 2.18)	−0.57 (−3.33; 2.19)
**Time in bed**	0.31 (−3.29; 3.91)	−0.45 (−4.07; 3.17)	−0.65 (−4.27; 2.98)

Adjusted R^2^ value for the crude model was −0.003, for the adjusted model 0.012 and for the fully adjusted model 0.137.

**Table A3:** Burnout.

Movement-Related Behavior	Crude Model	Adjusted Model	Fully Adjusted Model
	β (95% CI)	β (95% CI)	β (95% CI)
**MVPA**	−0.23 (−0.50; 0.04)	−0.25 (−0.52; 0.02)	−0.20 (−0.47; 0.07)
**LIPA**	−0.07 (−0.52; 0.38)	−0.04 (−0.49; 0.41)	0.02 (−0.43; 0.47)
**SED**	−0.24 (−0.79; 0.31)	−0.13 (−0.69; 0.44)	−0.21 (−0.78; 0.36)
**Time in bed**	0.54 (−0.22; 1.29)	0.42 (−0.35; 1.19)	0.39 (−0.37; 1.16)

Adjusted R^2^ value for the crude model was 0.002, for the adjusted model 0.005 and for the fully adjusted model 0.018.

**Table A4:** Mental wellbeing.

Movement-Related Behavior	Crude Model	Adjusted Model	Fully Adjusted Model
	β (95% CI)	β (95% CI)	β (95% CI)
**MVPA**	**7.01 *** **(1.16; 12.87)**	**7.1 *** **(1.20; 13.0)**	5.48 (−0.38; 11.34)
**LIPA**	−3.69 (−13.48; 6.11)	−3.72 (−13.57; 6.13)	−6.14 (−15.89; 3.61)
**SED**	−1.61 (−13.64; 10.42)	−3.66 (−16.01; 8.70)	−0.49 (−12.86; 11.86)
**Time in bed**	−1.72 (−18.25; 14.82)	0.28 (−16.52;17.08)	1.15 (−15.40; 17.70)

* Statistically significant associations are shown in bold and denoted by asterisks (*p* < 0.05). Adjusted R^2^ value for the crude model was 0.007, for the adjusted model 0.004 and for the fully adjusted model 0.04.

**Table A5:** Stress.

Movement-Related Behavior	Crude Model	Adjusted Model	Fully Adjusted Model
	β (95% CI)	β (95% CI)	β (95% CI)
**MVPA**	0.10 (−0.29; 0.50)	0.10 (−0.30; 0.50)	0.12 (−0.28; 0.52)
**LIPA**	−0.06 (−0.72; 0.60)	−0.07 (−0.73; 0.59)	−0.03 (−0.70; 0.64)
**SED**	−0.46 (−1.27; 0.35)	−0.27 (−1.10; 0.56)	−0.29 (−1.13; 0.56)
**Time in bed**	0.42 (−0.69; 1.54)	0.24 (−0.88; 1.37)	0.20 (−0.93; 1.32)

Adjusted R^2^ value for the crude model was 0.004, for the adjusted model 0.003 and for the fully adjusted model 0.002.

## Appendix C

**Table A6:** Predicted mental wellbeing scores and 95% CI for theoretically altered 24 h movement compositions. Time between MVPA and remaining behaviors were reallocated in steps of 10 minutes (one-for-one reallocations).

Reallocations	Compositional Mean of Daily Minutes Spent in MVPA	Predicted Mental Wellbeing Score after Theoretically Replacing MVPA with LIPA (95% CI)	Predicted Mental Wellbeing Score after Theoretically Replacing MVPA with SED (95% CI)	Predicted Mental Wellbeing Score after Theoretically Replacing MVPA with Time in Bed (95% CI)
**Theoretically reducing** **time spent in MVPA**	2 min	38 (20; 57)	39 (21; 57)	39 (20; 57)
	12 min	50 (41; 59)	50 (41; 59)	50 (41; 60)
	22 min	54 (47; 60)	54 (48; 60)	54 (48; 60)
	32 min	56 (51; 61)	56 (52; 61)	56 (52; 61)
	42 min	58 (54; 61)	58 (55; 61)	58 (55; 61)
	52 min	59 (56; 62)	59 (56; 62)	59 (56; 62)
**Samples average** **time spent in MVPA**	62 min	60 (58; 63)	60 (58; 63)	60 (58; 63)
**Theoretically increasing** **time spent in MVPA**	72 min	61 (59; 64)	61 (59; 64)	61 (59; 64)
	82 min	62 (59; 65)	62 (59; 65)	62 (59; 65)
	92 min	63 (60; 67)	63 (60; 66)	63 (59; 66)
	102 min	64 (60; 68)	64 (60; 67)	63 (59; 67)
	112 min	65 (60; 69)	64 (60; 68)	64 (59; 69)
	122 min	65 (60; 70)	65 (61; 69)	65 (59; 70)

MVPA: moderate to vigorous physical activity, LIPA: light intensity physical activity, SED: sedentary behavior.

## Appendix D

Results of the additional exploratory analysis for mental wellbeing where 24 h movement behavior was conceptualized as consisting of at least VPA, MPA, LIPA, SED and time in bed.

**Table A7:** *p*-Values from the ANOVA type II test of deviance of the regression, examining the association of the entire 24 h movement composition (consisting of at least VPA, MPA, LIPA, SED and time in bed) with mental wellbeing.

Mental Health Outcome	Crude Model	Adjusted Model	Fully Adjusted Model
Mental wellbeing	0.0059	0.0051	0.0963

Adjusted model includes covariates age, sex, education. Fully adjusted model additionally includes covariates sleep efficiency and fitness. At least VPA: at least vigorous physical activity, MPA: moderate physical activity, LIPA: light intensity physical activity, SED: sedentary behavior.

**Table A8:** *p*-Values, beta coefficients (95% CI) for the linear regression models assessing the association between relative time spent in each movement related behavior (at least VPA, MPA, LIPA, Sed, time in bed), relative to the remaining ones, and mental wellbeing. Table displays only results for the first *ilr* coordinate that contains all relative information about the respective behavior, relative to the geometric mean of the remaining ones.

Movement-Related Behavior	Crude Model		Adjusted Model		Fully Adjusted Model	
	*p*-Value	β (95% CI)	*p*-Value	β (95% CI)	*p*-Value	β (95% CI)
At least VPA	**0.0025 ***	**3.44** **(1.61; 5.28)**	**0.0027 ***	**3.49** **(1.63; 5.35)**	**0.0115 ***	**2.50** **(0.56; 4.44)**
MPA	0.8786	−0.49 (−6.73; 5.76)	0.9073	−0.37 (−6.64; 5.90)	0.8856	0.46 (−5.78; 6.69)
LIPA	0.8575	−0.87 (−10.44; 12.66)	0.8288	−1.06 (−10.66; 8.55)	0.4315	−3.87 (−13.51; 5.78)
SED	0.8637	1.02 (−10.63; 12.67)	0.8843	−0.85 (−12.82; 11.11)	0.8862	0.87 (−11.13; 12.88)
Time in bed	0.7005	−3.10 (−18.95; 12.74)	0.8829	−1.21 (−17.31; 14.89)	0.9973	0.03 (−15.94; 15.99)

* Statistically significant associations are shown in bold and denoted by asterisks (*p* < 0.05). Adjusted R^2^ value for the crude model was 0.028, for the adjusted model 0.025 and for the fully adjusted model 0.048.
